# Professional Quality of Life, Job Satisfaction, and Intention to Leave among Psychiatric Nurses: A Cross-Sectional Study

**DOI:** 10.3390/nursrep14020055

**Published:** 2024-03-26

**Authors:** Shaher Hamaideh, Abdallah Abu Khait, Hanan Al-Modallal, Rami Masa’deh, Ayman Hamdan-Mansour, Mohammed AlBashtawy

**Affiliations:** 1 Community and Mental Health Nursing Department, Faculty of Nursing, The Hashemite University, Zarqa 13133, Jordan; abdallah.abukhait@hu.edu.jo (A.A.K.); hmodallal@hu.edu.jo (H.A.-M.); 2 School of Nursing, Applied Science Private University, Amman 11937, Jordan; r_masadeh@asu.edu.jo; 3 Community Health Department, School of Nursing, The University of Jordan, Amman 11942, Jordan; a.mansour@ju.edu.jo; 4 Princess Salma Faculty of Nursing, Al al-Bayt University, Mafraq 25113, Jordan

**Keywords:** quality of life, job satisfaction, intention to leave, psychiatric nurses, Jordan

## Abstract

Psychiatric nurses are challenged with high levels of stress, which, in turn, lower their professional quality of life (ProQoL) and job satisfaction and increase their intention to leave jobs in psychiatric settings. An adequate level of ProQoL improves patient care provision. The purpose of this study is to assess the levels, relationships, and predictors of the professional quality of life of Jordanian psychiatric nurses and their job satisfaction and intention to leave their job. A descriptive cross-sectional design was used to collect data using the Professional Quality of Life Scale-5 from a convenience sample. Data were collected from 144 psychiatric nurses working in the largest psychiatric hospitals run by Ministry of Health, military, and university-affiliated hospitals. The results showed that the mean scores for compassion satisfaction (CS), burnout (BO), and secondary traumatic stress (STS) were 35.21, 27.0, and 26.36, respectively. The mean scores for intention to leave and job satisfaction were 2.33 and 3.7, respectively. The income level, educational level, intention to leave, and job satisfaction significantly differed in the CS dimension. Nurses’ age, intention to leave, and job satisfaction significantly differed in BO. Nurses’ age, years of experience, frequent C-shifts worked, and work affiliation were different in STS. Burnout, STS, and educational level predicted CS. CS, STS, job satisfaction, and level of education predicted BO. BO, CS, and frequently working night C-shifts predicted STS. Due to the presence of nurses reporting low levels of ProQoL, job satisfaction, and intension to leave, more studies are needed to assess the factors that affect ProQoL among psychiatric nurses and to evaluate interventional programs that improve the quality of life of psychiatric nurses. This is important in retaining nurses and increasing their job satisfaction. There is a need for interventions that enhance ProQoL. Also, longitudinal studies that measure ProQoL overtime are recommended. This study was not registered.

## 1. Introduction

Nurses in general, and psychiatric nurses in particular, work in a highly stressful and demanding environment. They work long and inconsistent hours, with low income, and deal with difficult patients, which may affect their wellbeing and job satisfaction and lead to burnout [1,2]. Psychiatric nurses experience more challenging and demanding work duties [3]. They care for difficult patients who cause burnout and fatigue [4]. Maintaining a good professional quality of life (ProQoL) for psychiatric nurses is very important for healthcare systems, in order to be able to retain qualified and experienced nurses, and for patient outcomes [5,6,7]. Psychiatric nurses working in Jordanian inpatient settings provide most of the psychiatric nursing tasks that reflect positively on patients and outcomes. Examples of these tasks include assessing and participating in physiological and psychological treatments [8]. With adequate ProQoL, psychiatric nurses provide adequate and comprehensive nursing care, which, in turn, improves patients’ outcomes. This subsequently leads to job satisfaction and therefore decreases the intention to leave the job [3,4,6].

ProQoL is described as the degree to which a group of workers are able to fulfil their personal needs through their experiences in their organizations [8]. ProQoL is composed of three related dimensions. Two of them are negative consequences of professional caregiving: compassion fatigue (CF) and burnout (BO). The third dimension is compassion satisfaction (CS), which is the positive consequences of professional care giving [8]. CF, which is sometimes called “secondary traumatic stress (STS)”, is defined as a natural outcome of physical and emotional reactions to caring for traumatized patients [8]. Nurses with high levels of STS experience physical and psychological effects such as fatigue, insomnia, headaches, abdominal disturbances, sadness, stress, depression, and decreases in attention and concentration [9,10,11,12]. BO is described as a feeling of difficulty in performing one’s work effectively [8]. Nurses suffering from BO exhibit many physical and emotional difficulties such as stress, anxiety, muscle itches, and job dissatisfaction and have high intention to leave their job [6,13]. CS is described as the pleasure and satisfaction that one feels when being able to provide effective care to people or patients [8]. In contrast to what nurses feel in BO and STS, nurses during CS feel more relaxed and satisfied with their job and have low intention to leave [14,15]. There are Jordanian studies available that measure job satisfaction and intention to leave among nurses, but none of them studied these concepts in relation to the professional quality of life of psychiatric patients.

There are several studies that have measured the levels and predictors of ProQoL among healthcare providers/professionals [16,17,18]; among nurses working in general hospitals [2,18,19,20,21]; and among mental health professionals [9,22]. Also, there are several studies that measure the levels and predictors of ProQoL among psychiatric nurses [5,11,13,14,23,24,25,26,27,28]. However, some of these studies measured only one dimension of the ProQoL. For example, Jalal et al. (2019) [29] measured secondary traumatic stress among psychiatric (STS) nurses, and found that the level was 37.64. Also, Abram and Jacobowitz (2021) [26] measured BO among psychiatric nurses and found the level to be 19.0.

The results for the levels of ProQoL among psychiatric nurses were varied in general and ranged from 23 to 43, 17 to 27, and 13 to 27 for CS, BO, and STS, respectively. For example, the lowest CS mean score (23.40) was reported by Iranian psychiatric nurses [27], and the highest (43.45) was reported by South African mental health nurses [24]. Regarding BO, the lowest mean score (17.46) was reported by Turkish psychiatric nurses [23], and the highest (27.17) was among Iranian psychiatric nurses [27]. Concerning STS, the lowest mean score (13.33) was found among Turkish mental health nurses [14], and the highest (27.36) was reported by South African psychiatric nurses [13]. ProQoL has been correlated with, and predicted by, many demographic and work-related variables. These variables include age, gender, marital status, income, educational level, years of experience in mental health settings, ward type (acute versus chronic), working shifts, intention to leave psychiatric units, and job satisfaction [5,11,13,14,23,24,25,26,27,28].

Intention to leave working at psychiatric settings has been documented in the literature [15,30,31,32,33,34,35]. Several factors have been associated with intention to leave work at psychiatric hospitals, especially in closed units. For example, Jiang et al. (2019) [33] found that more than 20% of Chinese psychiatric nurses had intention to leave their current jobs. Results have also found that psychiatric nurses with poor self-rated health, with a lower income level, dissatisfied with their job, and working more hours had a higher intention of leaving their job. A study conducted among Jordanian psychiatric nurses found that nurses who were male and single, with a Bachelor degree, and working in acute wards, had a higher intention of leaving their jobs [31]. Several personal and organizational factors affect psychiatric nurses’ job satisfaction. Examples of these factors include the work environment, intention to leave, age, gender, and education level [31]. Also, the intention to leave one’s job correlated negatively with job satisfaction [31] and with ProQoL [9].

There are no available Jordanian studies that assessed the ProQoL and its correlates among psychiatric nurses. Therefore, this study was conducted to assess the levels, correlates, and predictors of professional quality of life among Jordanian psychiatric nurses. More specifically, this study aimed to achieve the following:

1. Assess the levels of professional quality of life dimensions (CS, BO, and STS), job satisfaction, and intention to leave among psychiatric nurses;
2. Examine the relationships between professional quality of life dimensions (CS, BO, and STS), job satisfaction, and intention to leave among psychiatric nurses;
3. Detect differences (if any) in professional quality of life dimensions, job satisfaction, and intention to leave with selected demographic and work-related variables among psychiatric nurses;
4. Assess the predictors of professional quality of life dimensions (CS, BO, and STS) among psychiatric nurses.

## 2. Methods

A cross-sectional descriptive design was employed to assess the levels and predictors of professional quality of life among psychiatric nurses. The reporting of this research adheres to STROBE reporting guidelines for observational research [36].

### 2.1. Setting

Data were collected in June 2022 from psychiatric nurses working in inpatient mental healthcare settings in Jordan. Mental healthcare services in Jordan consists of four major sectors: Ministry of Health (MOH) sector, military sector, one private hospital, and two university-affiliated hospitals. Number of beds available for psychiatric patients in Jordan is about 677. Of them, 495 beds are run by MOH, 38 beds are run by the military sector, 120 beds are in a private hospital, and 24 are between two university-affiliated hospitals (World Health Organization [WHO], 2020). In Jordan, the total number of psychiatric nurses working in all mental healthcare hospitals is approximately 441 nurses. Most of them (268) are working in the MOH sector, 51 in the military sector, 86 in the private sector, and 36 in university hospitals (personal communication).

### 2.2. Sampling and Data Collection

G*Power equation was used to estimate the minimal required sample size. For a linear multiple regression with 13 predictors, a power of 0.80, an α of 0.05, with a small effect size (0.15), and a two-tailed test, the minimum sample size was recommended to be about 131 participants [37]. All Jordanian psychiatric nurses who had been working in inpatient psychiatric settings for at least one year were eligible to participate in the study. Nurses who do not provide direct patient care, such as administrators and supervisors, were excluded. In Jordan, the total number of nurses working in inpatient psychiatric settings is approximately 440 nurses. During the period of data collection, the number of eligible nurses was around 245. A total of 245 questionnaires were distributed to all the eligible psychiatric nurses. If they decided to participate in the study, psychiatric nurses were told to fill out the questionnaire and drop it in a sealed box placed in the unit’s reception. Sealed boxes were collected from all hospitals after two weeks of data collection. Completed questionnaires were received from 144 nurses (response rate = 58.7%).

### 2.3. Instruments

Data were collected using a questionnaire that contains four parts.

#### 2.3.1. Demographic and Work-Related Variables

Demographic variables were: age, gender, marital status (single, married), monthly income (by Jordanian Dinar [JOD]; JOD 1 equals USD 1.4), educational level (associate, bachelor, master, or higher), years of experience in mental health settings, ward (acute, chronic, or mixed cases), working shift (day—A, evening—B, night—C, or mixed—ABC), and setting type (Ministry of Health, Royal Military Medical Services, or educational hospital).

#### 2.3.2. Professional Quality of Life Scale, Version 5 (ProQoL-5)

ProQoL-5 which was developed by Stamm in 2010, was used to measure CS, BO, and STS among psychiatric nurses [6]. ProQoL-5 consists of 3 subscales (10 items each). It is a 5-point Likert-type scale ranging from 1 (never) to 5 (very often). For each subscale, the possible score ranges from 10 to 50. The higher the score, the higher the level of CS, BO, and STS. A score from 10 to 22 indicates a low level, a score from 23 to 41 indicates an average (moderate) level, and a score from 42 to 50 indicates a high level. The scale has good validity and reliability. Cronbach’s alphas were 0.88, 0.75, 0.81, and 0.88 for CS, BO, STS, and overall scale, respectively [6]. It has adequate convergent, discriminant, and construct validity [38]. This tool has been used to measure professional quality of life among Jordanian emergency nurses [39,40] and among Saudi Arabian psychiatric nurses [41]. The Arabic version of ProQoL-5 that was used in this study has been validated in Arabic Language [42]. In the current study, the Cronbach’s alphas were 0.81, 0.71, 0.80, and 0.76 for CS, BO, STS, and overall scale, respectively.

#### 2.3.3. Intention to Leave

Intention to leave psychiatric nursing was measured by asking the psychiatric nurses about their intention to stop working at psychiatric settings. The following statement was used: “If you have the chance to leave working in psychiatric settings, would you do that?” Responses were designed using a 5-Point-Likert-type scale, ranging from 1 = strongly disagree to 5 = strongly agree. Higher scores indicated higher intention to leave one’s job. Dolbeir et al. (2004) used a single item to measure job satisfaction, and it was found to be valid. Further, job satisfaction showed predictability for turnover intention [43].

#### 2.3.4. Job Satisfaction

Job satisfaction was measured by asking the nurses about their level of satisfaction in working in psychiatric setting using the following statement: “Considering all things, how satisfied are you working in a psychiatric setting?” Responses were designed using a 5-Point-Likert-type scale, ranging from 1 = strongly dissatisfied to 5 = strongly satisfied. Higher scores indicated higher job satisfaction. Kivimaki et al. (2007) used a single item to measure the intention to leave among hospital employees and found it to be valid [44].

### 2.4. Ethical Considerations

Ethical approval was obtained from the university’s Intuitional Review Board (IRB) (No: 18/6/2021/2022). All psychiatric nurses who agreed to participate were told to sign an attached consent form with each questionnaire. The consent form includes all information regarding the study’s purpose, instructions, and freedom to withdraw at any time. Nurses were told that their participation was voluntary, and no personal or identifying information would be collected. Anonymity was assured by not asking for any personal or identification data. To assure the confidentiality of data, the questionnaires were handled only by the primary investigator and data were stored in a personal computer with a password. Participants were told that they may experience very minimal potential psychological discomfort while completing the questionnaire. They were told to stop filling out the questionnaire if they felt any discomfort.

### 2.5. Statistical Analysis

The Statistical Package for Social Sciences (SPSS) version 22 was used to analyze data. Descriptive statistics were employed to measure frequencies, percentages, means, and standard deviations. Relationships between variables were examined using either Pearson correlation coefficients (for continuous variables) or Spearman rho (for categorial variables). Linear multiple regression analyses were run to detect the variables that best predict ProQoL dimensions separately (CS, BO, and STS) and the intention to leave one’s job. Significance level was at 0.05.

## 3. Results

### 3.1. Description of Demographic and Work-Related Variables

The final sample was composed of 144 psychiatric nurses; 65 (45.1%) were females, 115 (79.9%) were married, and 98 (68.1%) held a bachelor degree. Their age ranged between 21 and 51 years (mean = 35.61, SD ± 6.55). Psychiatric nurses’ experience in psychiatric settings ranged between 2 and 30 years (mean = 11.71, SD ± 7.93). Regarding their intention to stop working in psychiatric settings, 97 (67.3%) strongly disagreed/disagreed with leaving and 35 (24.4%) strongly agreed/agreed with leaving, while 12 (8.3%) were neutral toward their intention to leave psychiatric settings. Regarding job satisfaction, 23 (16.0%) were strongly dissatisfied/dissatisfied with their job and 103 (71.6%) were strongly satisfied/satisfied with their job, while 18 (12.4%) were neutral regarding their job satisfaction. Other demographic and work-related variables are shown in Table 1.

### 3.2. Levels of Dimensions of Professional Quality of Life, Intention to Leave, and Job Satisfaction

The mean score of professional quality of life dimensions, CS, BO, and STS, were 35.21 (SD ± 6.06), 27.0 (SD ± 4.24), and 26.36 (SD ± 6.44), respectively. The frequencies and percentages of low, moderate, and high levels of CS, BO, and STS are presented in detail in Table 2. The highest percentages of ProQoL dimensions (CS, BO, and STS) were moderate. On a scale from 1 to 5, the mean score for intention to leave was 2.33 (SD ± 1.34), indicating a low level. The mean score for job satisfaction was 3.7 (SD ± 0.99), indicating a high level (see Table 2).

### 3.3. Relationships of Professional Quality of Life Dimensions, Intention to Leave, and Job Satisfaction

Compassion satisfaction correlated positively with monthly income (r = 0.295, *p* < 0.001), educational level (r = 0.270, *p* < 0.000), and job satisfaction (r = 0.347, *p* < 0.001). CS correlated negatively with BO (r = −0.528, *p* < 0.001), STS (r = −0.179, *p* < 0.05), and intention of leaving psychiatric settings (r = −0.199, *p* < 0.05). Burnout correlated positively with STS (r = 0.351, *p* < 0.001) and with the intention to leave (r = −0.196, *p* < 0.05), and negatively with job satisfaction (r = −0.331, *p* < 0.001). Secondary traumatic stress correlated positively with frequently worked shift (r = 0.226, *p* < 0.001) and with place of work (r = 0.190, *p* < 0.05) (see Table 3).

### 3.4. Differences in Professional Quality of Life Dimensions with Demographics

There are differences in income level, educational level, intention to leave, and job satisfaction in compassion satisfaction dimensions. A higher CS was found among those who had no intention to leave and among those who were satisfied. Also, post hoc analysis revealed that nurses with an income level of JOD 640–900 reported a higher level of CS compared to those with income levels of JOD 400–599 and 600–639. Also, psychiatric nurses with bachelor and master degrees had higher CS than those with associate degrees.

Regarding BO, psychiatric nurses’ age, intention to leave, and job satisfaction were significantly different. BO was higher among those aged 21–36 than those aged 36–51. Also, BO was higher among those who had an intention to leave and those who were dissatisfied with their job. In terms of STS, differences were found in relation to psychiatric nurses’ age, years of experience, frequent night (C) shift worked, and work affiliation. STS was higher among younger nurses (aged 21–36), less experienced nurses (working for 1–9 years), those working the night (C) shift regularly, and those affiliated with teaching university hospitals (see Table 4).

### 3.5. Predictors of Professional Quality of Life Dimensions

Three separate multiple regression analyses were performed to detect the variables that predict ProQoL dimensions (CS, BO, and STS). In each analysis, all demographic and work-related variables were entered as independent variables. Burnout, STS, and educational level predicted CS and accounted for 47.7% of the total variance. CS, STS, job satisfaction, and level of education predicted BO and accounted for 51.2% of the total variance. BO, CS, and frequent working the night (C) shift predicted STS and accounted for 32.3% of the total variance (see Table 5).

## 4. Discussion

The primary aims of the current study were to assess the levels, relationships, and predictors of the professional quality of life of Jordanian psychiatric nurses, as well as to assess the correlations of quality of life dimensions with psychiatric nurses’ job satisfaction and intention of leaving their job.

### 4.1. Levels of Professional Quality of Life, Intention to Leave, and Job Satisfaction

The results of the current study indicate that the levels of CS, BO, and STS were 35.21, 27.0, and 26.36, respectively, reflecting moderate levels. Also, the results show that the mean score for intention to leave and job satisfaction were 2.33 and 3.7, respectively. These results are congruent with the results found in most similar studies that have measured CS, BO, and STS among psychiatric nurses. In several studies, the CS level has been found to be between 20.0 [5] and 41.0 [13], indicating a moderate level. For example, among 122 South Korean psychiatric nurses, CS was 35.05 [25], and it was 39.69 among UK psychiatric nurses [23]. Regarding BO, most similar studies reported the level of BO among psychiatric nurses to be between 17.0 [26] and 40.0 [5], indicating a moderate level. For instance, the BO level among 160 psychiatric Iranian nurses was 27.17 [27], and it was 26.92 among 352 Chinese psychiatric nurses [28]. Concerning STS, most studies that measure STS among psychiatric nurses found the results to be between 13 [14] and 27.0 [13], indicating a moderate level. For examples, the STS among 163 South African psychiatric nurses was 27.36 [10], and it was around 26.0 among 352 Chinese psychiatric nurses [28].

The results show that the mean score for intention to leave was 2.33 (lower than the midpoint, which is 2.5) and the mean score for job satisfaction was 3.7 (higher than the midpoint, which is 2.50). These results are congruent with results found by Zheng et al., (2017) that showed that most psychiatric nurses were satisfied with their jobs, with most job satisfaction items ranging between 3.03 and 3.79 out of 5.0. Further, Baum and Kagan (2015) [15] found that the mean score for psychiatric nurses’ job satisfaction was 3.8 out of 5. Also, our results regarding intention to leave are congruent with the results found by Kagwe et al. (2019) [31], who found that around one third of psychiatric nurses had intention to leave for another employment. However, contrary to our results, Jiang et al. (2019) [33] found a high percentage of psychiatric nurses who intended to leave their current job.

### 4.2. Relationships among Professional Quality of Life, Intention of Leaving, and Job Satisfaction

The results indicate that the CS correlated positively with monthly income, educational level, and job satisfaction and negatively with BO, STS, and intention to leave psychiatric settings. BO correlated positively with STS and intention to leave and negatively with job satisfaction. STS correlated positively with frequent working shift and place of work. Our results regarding the relationships among CS, BO, and STS were congruent with the results of most studies that measure the quality of life among psychiatric nurses. For instances, several studies found that CS correlated negatively with BO and STS and BO correlated positively with STS [14,22,26]. In line with our results regarding job satisfaction, Emmanuel and Odusanya (2015) [45] found that psychological wellbeing and general health correlated positively with job satisfaction.

### 4.3. Differences in Professional Quality of Life with Demographics

The results of this study found that CS is higher among those who are satisfied, have no intension to leave, have a higher income, and have a higher educational level. The results also indicate that BO is higher among younger nurses, those who have an intention of leaving, and those who are dissatisfied with their job. Further, the results of the current study show that STS is higher among younger, less experienced nurses, working the C shift regularly, and those who are affiliated with teaching university hospitals. Our results are consistent with the results of other studies that found differences in demographics in relation to CS, BO, and STS. For instance, Mangoulia et al. (2015) [11] found differences in CS, BO, and STS among 174 psychiatric nurses working in psychiatric units regarding their willingness to leave their current job. In addition, Basogul et al. (2021) [14] found that CS was higher among those who had no intention of leaving.

With regard to quality of life and educational level, Alhawatmeh et al., (2021) [1] found that the quality of life of Jordanian nurses was higher among nurses with a higher level of educational (master degree). However, Park (2021) [25] and Xie et al. (2020) [28] found that nurses with a diploma had higher levels of BO and STS. Regarding working shifts, Ruiz-Fernandez et al. (2020) [21] found that nurses who were mostly working in the evening reported higher levels of STS, which is consistent with the results found in the current study. Our results are also consistent with the results of Xie et al. (2020) [28], who found that BO and STS were higher among younger psychiatric nurses. Also, Ruiz-Fernandez et al. (2021) [22] found that CS was higher among younger psychiatric nurses. Further, Xie, at al. (2020) [28] found that less experienced nurses had higher levels of STS, which supports our results. Contrary to the finding in psychiatric nurses that CS, BO, and STS were different according to the gender [20,26,28], our results found no differences between male and female nurses with regard to their dimensions of quality of life. Female nurses reported higher levels of CS and STS [11,22,28].

### 4.4. Predictors of Professional Quality of Life

In the current study, BO, STS, and educational level predicted CS; CS, STS, job satisfaction, and level of education predicted BO; and BO, CS, and frequent working shift predicted STS. Our results regarding the predictor variables are consistent with the results found by Park (2021) [25], who reported that educational level predicted BO and STS. Also, similar findings were reported by Xie et al. (2020) [28], who found that job satisfaction predicted CS, BO, and STS among psychiatric nurses. Further, in a sample of Iranian doctors, nurses, and midwives, Keshavarz et al. (2019) [16] found that job satisfaction, older age, and monthly income predicted CS; job satisfaction, working night shifts, and less experience predicted BO. Also, STS was predicted by job satisfaction, female gender, working the C shift, and income level [16,21].

### 4.5. Limitations

This study is the first study that assess the levels and correlations of ProQoL among Jordanian psychiatric nurses working in inpatient psychiatric units. However, there are some limitations to this study. First, the use of a cross-sectional design allowed us to establish associations among variables but did not permit the determination of causality among variables. Second, there is a risk of social desirability responses due to the use of self-reported data, which may affect the results. Third, the data were collected from nurses working in three psychiatric sectors (public, military, and university-affiliated) and did not include nurses working in the private sector. Therefore, the results cannot be generalized to psychiatric nurses working in the private sector. Finally, job satisfaction and intention of leaving were measured via a single question with responses ranging from 1 to 5, which did not give details about those concepts. Therefore, future research should be directed toward conducting longitudinal studies to explore changes in ProQoL overtime. Also, future research should investigate the impact of factors that were not covered in this study.

### 4.6. Implications

Future research studies should involve longitudinal studies to determine how these variables change over time and may involve psychiatric nurses working in private psychiatric hospitals. Future studies may employ more valid and reliable scales that measure job satisfaction and intention to leave the job. Also, future studies should explore more individual and institutional factors that influence the ProQoL. Further, we recommend interventional studies targeting the ProQoL of psychiatric nurses.

Continuing educational programs on ProQoL to enhance CS and reduce BO and STS are recommended to improve psychiatric nurses’ psychological wellbeing, job satisfaction, and retention. Stress reduction programs may also help to improve ProQoL especially for younger and less experienced nurses. Psychiatric nurses also need to incorporate some type of professional self-care activities (e.g., yoga, exercise, recreational activities) in their professional life to buffer the effects of BO and STS. It is essential to perform ongoing assessment and evaluation for psychiatric nurses’ ProQoL, job satisfaction, and intention to leave. Further, stress reduction programs and continuing education initiatives should be conducted to improve ProQoL among psychiatric nurses.

## 5. Conclusions

This study aimed to assess the levels, relationships, and predictors of professional quality of life of Jordanian psychiatric nurses and their job satisfaction and intention to leave their job. The results of this study show that the mean scores for compassion satisfaction (CS), burnout (BO), and secondary traumatic stress (STS) were 35.21, 27.0, and 26.36, respectively. Further, the mean scores for intention to leave and job satisfaction were 2.33, and 3.7, respectively. This study also affirmed the strong relationships between ProQoL dimensions. Strong relationships between these variables can inform interventions and support strategies that enhance patients’ outcomes. CS was higher among nurses with a higher income level, higher educational level, lower intention to leave, and higher job satisfaction. BO was higher among younger nurses and nurses with a higher intention to leave and lower job satisfaction. STS was higher among younger and less experienced nurses, those working the C shift frequently, and those working in hospitals affiliated with universities. Several variables predicted the ProQoL dimensions. Recognizing these predictors can assist healthcare institutions in creating environments that promote nurses’ wellbeing. In addition, adequate levels of ProQoL and job satisfaction and low levels of intention to leave the job among psychiatric nurses improve nurses’ wellbeing and enhance psychiatric nursing practices. Further, higher levels of ProQoL among psychiatric nurses can positively impact the quality of care provided to psychiatric patients. Interventional strategies and continuous education aimed at enhancing CS and reducing BO and STS among psychiatric nurses are essential. Researchers are encouraged to build on this study’s findings to conduct more related studies. Finally, administrators and policy-makers should underscores the importance of prioritizing nurses’ wellbeing in psychiatric care settings.

## Figures and Tables

**Table 1:** Demographic and work-related variables (N = 144).

Variable	Mean	Standard Deviation
Age (years)	35.61	6.55
Monthly income (JD: Jordanian Dinar)	613.68	105.22
Experience in psychiatric settings (years)	11.72	7.93
	Frequencies	Percentages
Gender		
Female	65	45.1
Male	79	54.9
Marital status		
Single	29	20.1
Married	115	79.9
Educational level		
Associate	38	26.3
Bachelor	98	68.1
Master and more	8	5.6
Ward/unit		
Acute	69	47.9
Chronic	19	13.2
Mixed (acute and chronic)	56	38.9
Frequent working shift		
A—day	44	30.6
B—evening	39	27.1
C—night	61	42.4
Place of work		
Ministry of Health	70	48.6
Royal Medical Services	36	25.0
University Hospitals	38	26.4
Intent to leave psychiatric nursing		
Strongly disagree	49	34.0
Disagree	48	33.3
Neutral	12	8.3
Agree	21	14.7
Strongly agree	14	9.7
Job satisfaction		
Strongly dissatisfied	4	2.8
Dissatisfied	19	13.2
Neutral	18	12.4
Satisfied	78	54.2
Strongly Satisfied	25	17.4

**Table 2:** Mean scores for compassion satisfaction, burnout, and secondary traumatic stress (N = 144).

Scale	Possible Range	Mean	Standard Deviation
Compassion satisfaction	10–50	35.21	6.06
Burnout	10–50	27.00	4.24
Secondary traumatic stress	10–50	26.36	6.44
Intention of leaving nursing	1–5	2.33	1.34
Job satisfaction	1–5	3.70	0.99
		Frequency	Percentage
Compassion satisfaction			
Low	10–22	6	4.2
Moderate	23–41	116	80.5
High	42–50	22	13.3
Burnout			
Low	10–22	17	11.8
Moderate	23–41	125	86.8
High	42–50	2	1.4
Secondary traumatic stress			
Low	10–22	44	30.6
Moderate	23–41	97	67.3
High	42–50	3	2.1
Intention of leaving nursing			
Yes		35	26.5
No		97	73.5
Job satisfaction			
Satisfied		103	81.7
Not satisfied		23	18.3

**Table 3:** Associations of compassion satisfaction, burnout, and secondary traumatic stress with demographics (N = 144).

Variable	Compassion Satisfaction	Burnout	Secondary Traumatic Stress
Compassion satisfaction	1.00		
Burnout	−0.528 **	1.00	
Secondary traumatic stress	−0.179 *	0.351 **	1.00
Age	0.035	−0.115	−0.112
Gender	0.002	−0.069	−0.046
Marital status	0.135	−0.127	0.002
Monthly income	0.295 **	−0.145	0.023
Educational level	0.270 **	−0.050	−0.010
Experience in psychiatric settings	−0.023	−0.028	−0.115
Ward/unit	−0.021	−0.079	0.002
Frequent working shift	−0.076	0.146	0.226 **
Place of work	0.020	0.071	0.190 *
Intention of leaving psychiatric nursing	−0.199 *	0.196 *	−0.037
Job satisfaction	0.347 **	−0.331 **	0.081

* Significance at a two-tailed alpha of 0.05, ** Significance at a two-tailed alpha of 0.001.

**Table 4:** Differences in compassion satisfaction, burnout, and secondary traumatic stress with some demographics (N = 144).

Variable	Compassion Satisfaction			Burnout			STS		
	Mean	F/t	*p*	Mean	F/t	*p*	Mean	F/t	*p*
Age		−0.574	0.567		2.35	0.020		2.41	0.017
21–35	34.92			27.79			27.61		
36–51	35.51			26.16			25.06		
Income level		6.73	0.002		1.67	0.191		0.871	0.421
400–599	33.61			27.37			25.57		
600–639	34.52			27.52			26.38		
640–900	37.67			26.06			27.28		
Psychiatric experience		−0.133	0.894		1.82	0.070		2.03	0.044
1–9	35.15			27.67			27.49		
10–20	35.28			26.39			25.34		
Gender		−0.027	0.978		0.828	0.409		0.546	0.586
Female	35.20			27.32			26.69		
Male	35.22			26.73			26.10		
Marital status		−1.63	0.106		1.52	0.129		−0.022	0.983
Single	33.58			28.07			26.34		
Married	35.62			26.7			26.37		
Educational level		5.96	0.003		1.95	0.145		0.906	0.406
Associate	32.45			26.84			25.94		
Bachelor	36.10			27.28			26.74		
Master and more	37.50			24.25			23.75		
Ward/unit		0.332	0.718		1.13	0.327		0.528	0.591
Acute	35.20			27.52			26.16		
Chronic	36.21			26.10			27.78		
Mixed	34.89			26.66			26.14		
Frequent shift worked		1.43	0.242		1.64	0.198		3.86	0.023
A—day	36.34			26.07			24.59		
B—evening	34.10			27.18			25.84		
C—night	35.11			27.55			27.98		
Affiliation		0.032	0.969		2.15	0.120		4.72	0.010
Ministry of Health	35.08			27.01			25.70		
Royal Medical Services	35.30			25.94			24.89		
University Hospitals	35.36			27.97			29.00		
Intention of leaving nursing		2.94	0.004		−3.01	0.007		0.164	0.866
Yes	32.57			28.86			26.34		
No	35.94			26.38			26.56		
Job satisfaction		−3.89	<0.000		3.31	0.001		−1.46	0.146
Yes, satisfied	36.41			26.29			26.97		
No, not satisfied	31.13			29.48			24.74		

**Table 5:** Stepwise multiple regression model predicting compassion satisfaction, burnout, and secondary traumatic stress (N = 144).

	B	β	*F*	*p*	Confidence Interval (Lower, Upper)	
**Predictors of compassion satisfaction**			44.40	<0.000		
1. Burnout	−0.944	−0.661			−1.07	−0.65
2. Secondary traumatic stress	−0.388	0.413			0.24	0.50
3. Educational level	2.77	0.241			0.42	3.64
Model summary						
R^2^	0.488					
Adjusted R^2^	0.477					
Total variance	47.7%					
**Predictors of burnout**			38.53	<0.000		
1. Compassion satisfaction	−0.409	−0.585			−0.49	−0.30
2. Secondary traumatic stress	0.310	0.471			0.23	0.39
3. Job satisfaction	−0.749	−0.176			−1.19	0.002
4. Level of education	1.021	0.127			−0.04	2.16
Model summary						
R^2^	0.526					
Adjusted R^2^	0.512					
Total variance	51.2%					
**Predictors of secondary traumatic stress**			23.75	<0.000		
1. Burnout	0.898	0.591			0.72	1.23
2. Compassion satisfaction	−0.536	0.504			0.35	0.72
3. Frequent shift work	1.352	0.178			0.10	2.42
Model summary						
R^2^	0.337					
Adjusted R^2^	0.323					
Total variance	32.3%					

## Data Availability

Data are available from the corresponding author upon request.

## Public Involvement Statement

Psychiatric nurses were involved. They completed a self-administered questionnaire.

## Guidelines and Standards Statement

This manuscript was drafted using the STROBE guidelines for a cross-sectional study with descriptive research.
